# The mystery of the hidden gallbladder: Navigating the unseen

**DOI:** 10.1002/ccr3.9532

**Published:** 2024-10-29

**Authors:** Momin Shakeeb, Romana Riyaz, Sajjad Ahmed Khan, Khadija Fatima, Husna Fatima

**Affiliations:** ^1^ Owaisi Hospital and Research Centre Hyderabad Telangana India; ^2^ Shadan Institute of Medical Sciences and Research Hyderabad Telangana India; ^3^ Birat Medical College Teaching Hospital Morang Nepal; ^4^ Osmania Medical College Hyderabad Telangana India

**Keywords:** cholecystectomy, congenital anomaly, duplex gall bladder, hypothyroidism, radiodiagnosis

## Abstract

This case underscores the importance of advanced imaging techniques in diagnosing complex biliary anomalies. Accurate Magnetic Resonance Cholangiopancreatography and ultrasound evaluations can distinguish between choledochal cysts and duplicate gallbladders, guiding effective surgical intervention and improving patient outcomes.

## 1 INTRODUCTION

Biliary anomalies, including choledochal cysts and duplicate gallbladders, are rare conditions that pose significant diagnostic challenges and require precise imaging for effective management. Choledochal cysts are congenital dilations of the bile duct, with varying presentations and potential for serious complications if misdiagnosed or left untreated. These cysts are typically classified into five types based on their anatomical features, with Type I being the most common, characterized by a cystic dilation of the extrahepatic bile duct.^1^ Symptoms often include abdominal pain, jaundice, and recurrent cholangitis, which can mimic other biliary conditions and obscure the diagnosis.^1^ If not addressed, choledochal cysts can lead to severe complications such as biliary obstruction, pancreatitis, and even cholangiocarcinoma.^2^ Duplicate gallbladders, although extremely rare, present a unique challenge due to their similarity in presentation to other biliary disorders. This anomaly involves the presence of two distinct gallbladders, each with its own cystic duct and possibly its own blood supply. The clinical symptoms can overlap with those of choledochal cysts or other gallbladder pathologies, such as cholecystitis or choledocholithiasis, making accurate diagnosis crucial.^3^ The rarity of this condition means it is often not initially considered, leading to potential misdiagnosis or delayed treatment.

Imaging techniques are essential for differentiating between these conditions. Magnetic Resonance Cholangiopancreatography (MRCP) is particularly valuable for visualizing the biliary tree and identifying the specific characteristics of choledochal cysts and duplicate gallbladders. MRCP provides detailed images of the bile ducts and surrounding structures without the need for invasive procedures. It can clearly show the cystic dilation of choledochal cysts and help identify the anatomical configuration of duplicate gallbladders.^4^ Similarly, ultrasound is a first‐line imaging modality that can reveal the presence of multiple gallbladders, their anatomical relationships, and associated complications such as gallstones or inflammation.^5^

In this case, the use of MRCP and ultrasound played a critical role in accurately diagnosing a double gallbladder anomaly, which is a rare and complex condition. The MRCP findings included a normal‐appearing gallbladder and a thin‐walled cystic lesion adjacent to it, which demonstrated communication with the gallbladder. The presence of numerous calculi within the cystic lesion was also noted. Further imaging with B‐mode and 3‐dimensional (3D) ultrasound revealed a thin communicating tract between the gallbladder and the common bile duct, effectively ruling out a choledochal cyst and confirming the presence of a duplicate gallbladder. The accurate diagnosis of a duplicate gallbladder through advanced imaging allowed for appropriate surgical planning. The patient underwent an open cholecystectomy, during which the double gallbladder Y‐type anomaly was confirmed. The surgical findings included a distended extrahepatic gallbladder filled with clear mucus and a firm intrahepatic gallbladder containing multiple stones. The cystic duct was found to be short and straight, joining directly with the common bile duct. The presence of two cystic arteries and thick fibrofatty tissue at the hilum indicated acute chronic inflammation.

This case highlights the critical role of advanced imaging techniques in diagnosing complex biliary anomalies. Accurate MRCP and ultrasound evaluations enable clinicians to differentiate between choledochal cysts and duplicate gallbladders, leading to appropriate management and improved patient outcomes. The integration of these imaging modalities in clinical practice is essential for the effective diagnosis and treatment of rare and challenging biliary conditions.

## 2 CASE HISTORY/EXAMINATION

**FIGURE 1:**
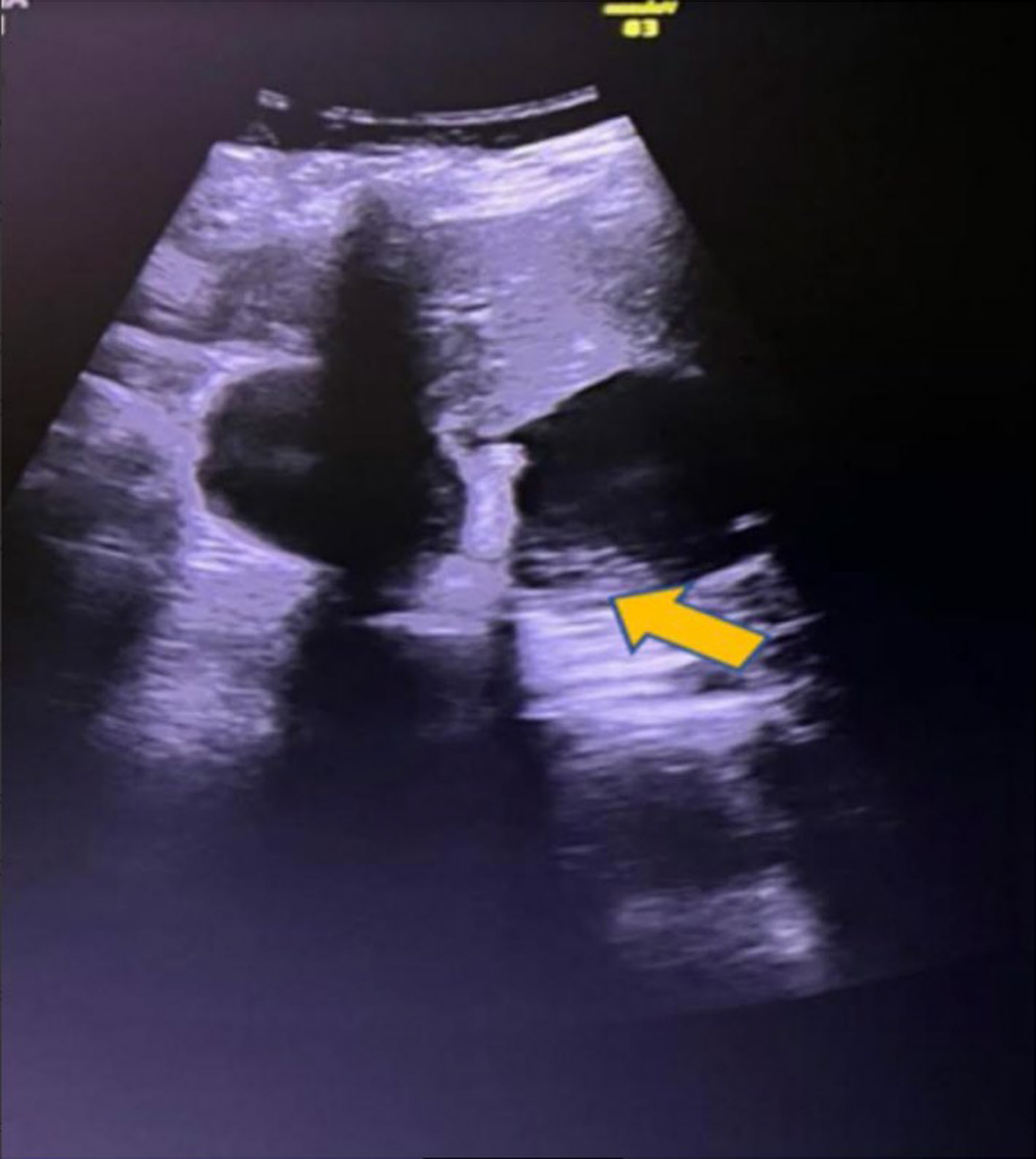
USG scan shows normal gallbladder and cystic lesion with multiple echogenic debris.

A 38‐year‐old female presented with a three‐month history of recurrent, severe right upper quadrant abdominal pain. The pain was intense and frequent, accompanied by non‐bilious vomiting, persistent nausea, and significant weight loss. The patient had a known history of hypothyroidism and was receiving levothyroxine treatment (75 μg daily). Despite this, there was no family history of similar symptoms or liver disease. On examination, the patient appeared anemic and icteric, suggesting hepatic dysfunction. Tenderness was particularly noted in the epigastric region. Laboratory tests revealed mild elevation in liver enzymes, including alanine aminotransferase and aspartate aminotransferase, indicating possible liver inflammation or dysfunction. Mild conjugated hyperbilirubinemia suggested possible biliary obstruction or hepatic impairment. Mild hypercholesterolemia was also noted. An abdominal ultrasound scan showed a normal gallbladder but identified a cystic lesion in the right lobe of the liver. This lesion measured 7 × 3 cm and had irregular borders with echogenic debris, raising concerns about a possible hepatic or biliary pathology (Figure 1).

## 3 METHODS (DIFFERENTIAL DIAGNOSIS, INVESTIGATIONS, AND TREATMENT)

**FIGURE 2:**
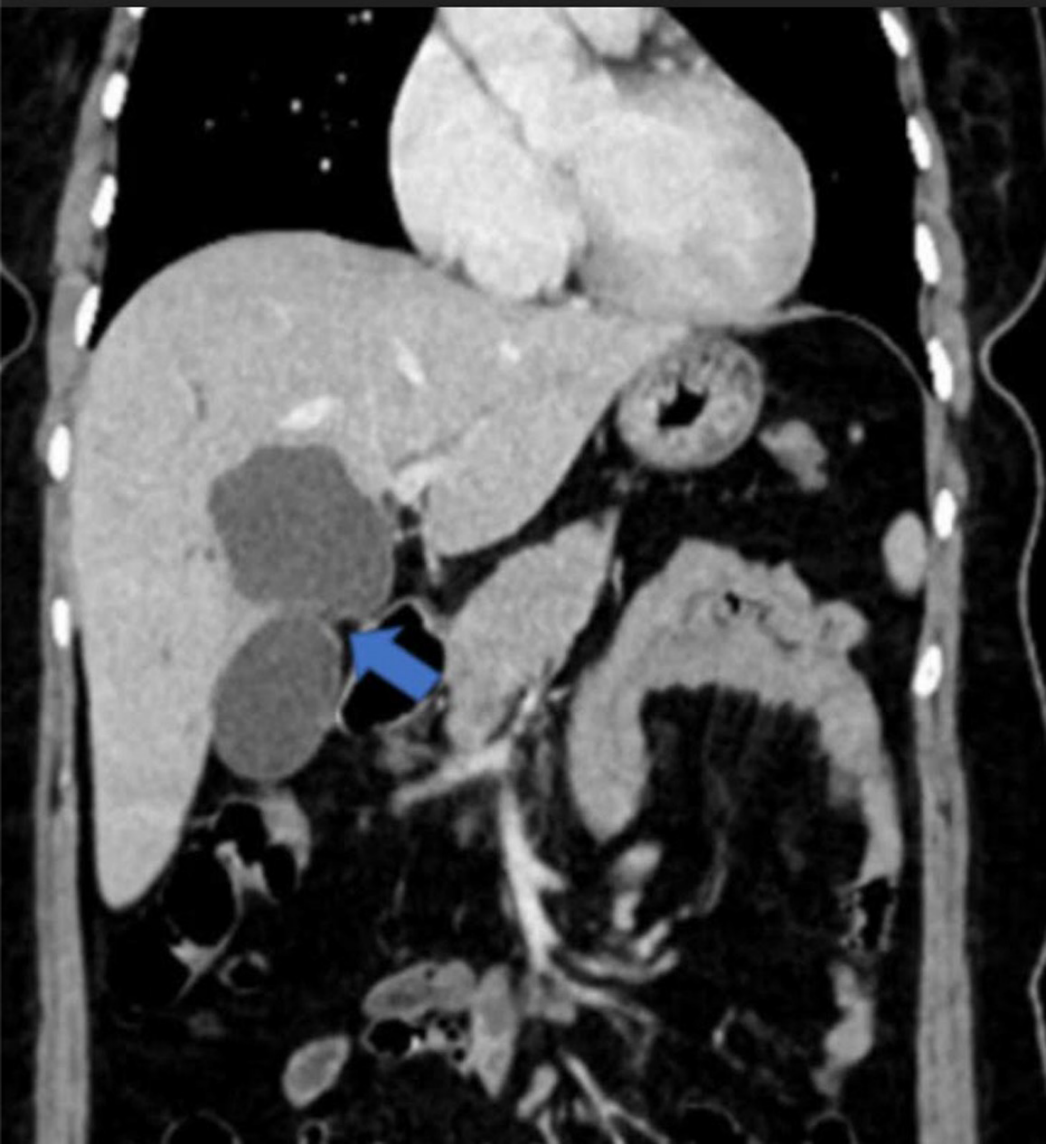
CT coronal section in venous phase shows intrahepatic cystic lesion.

**FIGURE 3:**
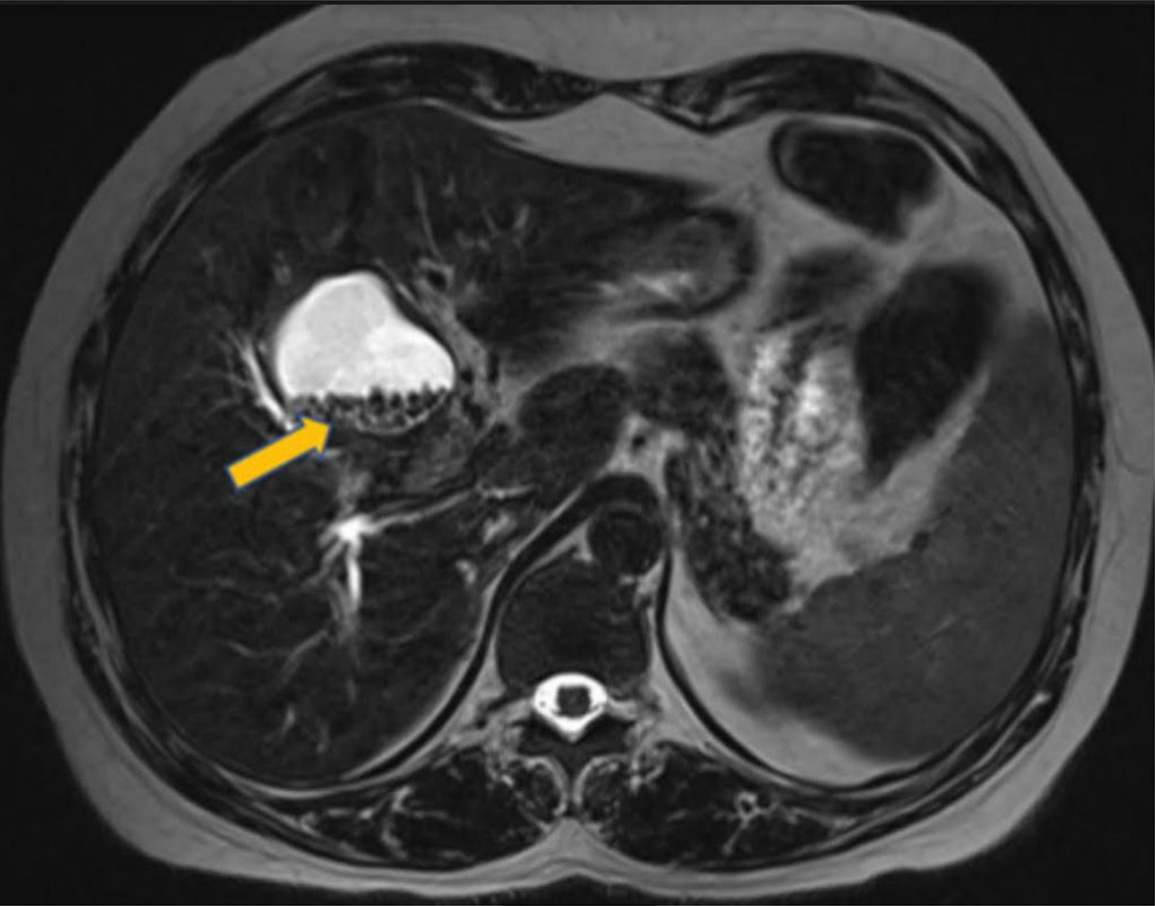
MRCP Coronal T2 HASTE sequences shows a Y shaped double gall bladder.

**FIGURE 4:**
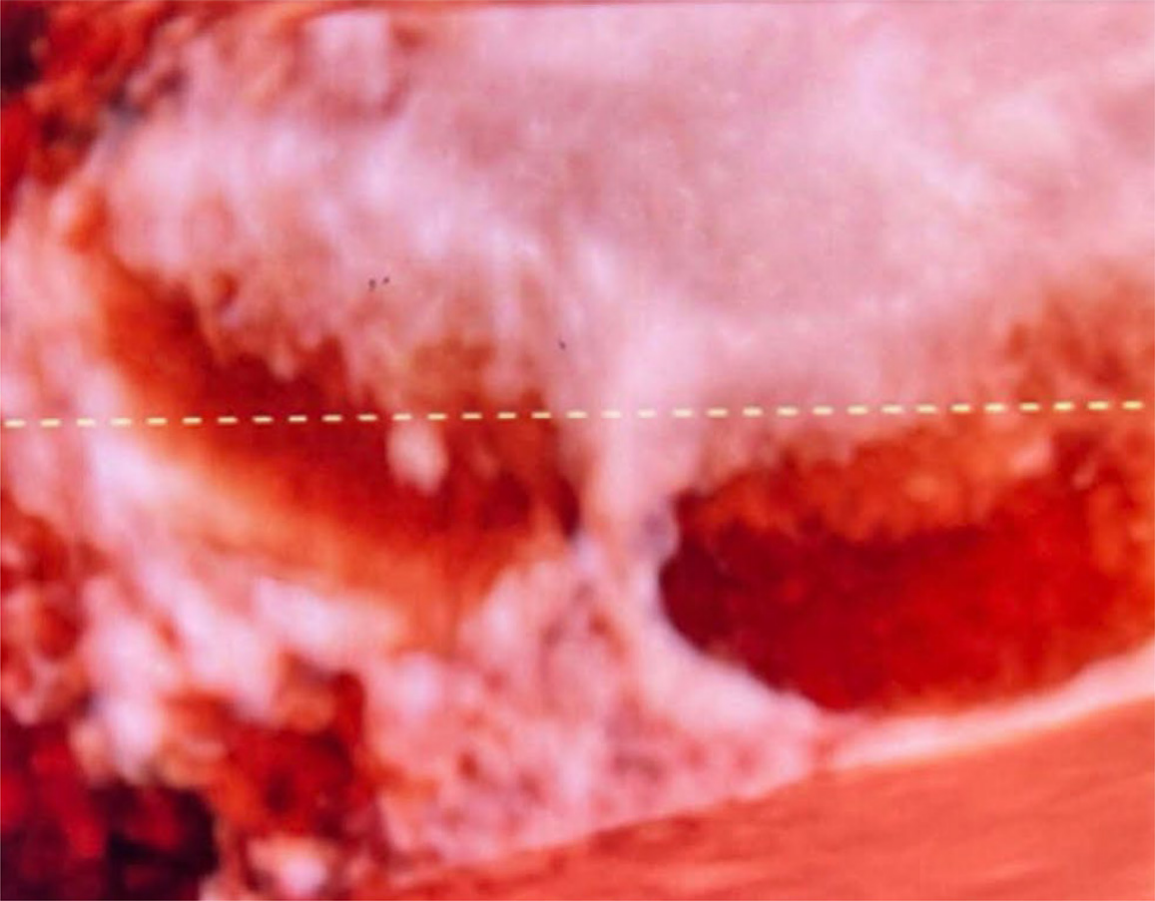
B mode and 3D mode USG scans shows normal common bile duct.

**FIGURE 5:**
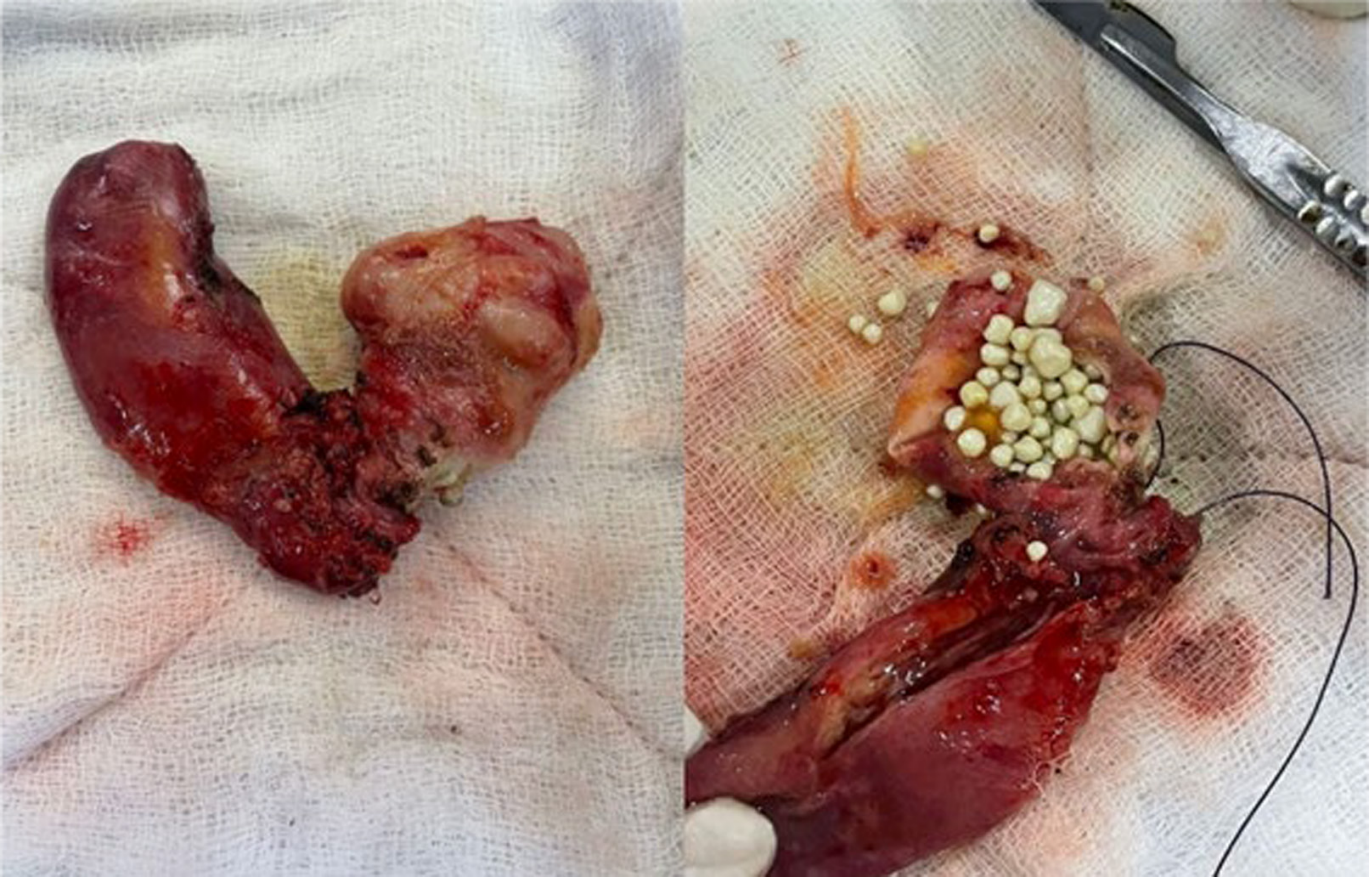
Specimen of Y shaped double gall bladder.

To differentiate between potential diagnoses, such as choledochal cysts or a duplicate gallbladder, further imaging was conducted using Computed Tomography (CT) and MRCP. CT coronal section in venous phase shows intrahepatic cystic lesion (Figure 2). MRCP with T2 HASTE sequences provided detailed images of the abdominal structures. The results showed a normal gallbladder with no abnormalities in the wall. Adjacent to the gallbladder, a thin‐walled cystic lesion was observed. This lesion was hyperintense on T2‐weighted images, indicating high fluid content. Importantly, the lesion demonstrated a T2 hyperintense communication with the gallbladder, suggesting a direct connection between the two structures. Additionally, numerous T2 hypointense calculi were present within the cystic lesion, indicating the presence of stones or calcified material (Figure 3). Further imaging with B‐mode and 3D ultrasound revealed a thin communicating tract between the gallbladder and the common bile duct, effectively ruling out a choledochal cyst as the diagnosis (Figure 4). For definitive diagnosis and treatment, the patient underwent open cholecystectomy under general anesthesia. During the procedure, a double gallbladder Y‐type anomaly was identified. The findings included a distended extrahepatic gallbladder filled with clear mucus and a firm intrahepatic gallbladder containing multiple stones. The cystic duct was short and straight, joining directly with the common bile duct, and was fully dissected. Two cystic arteries were found with thick fibrofatty tissue at the hilum, indicative of acute on chronic inflammation (Figure 5).

## 4 CONCLUSION AND RESULTS (OUTCOME AND FOLLOW‐UP)

**FIGURE 6:**
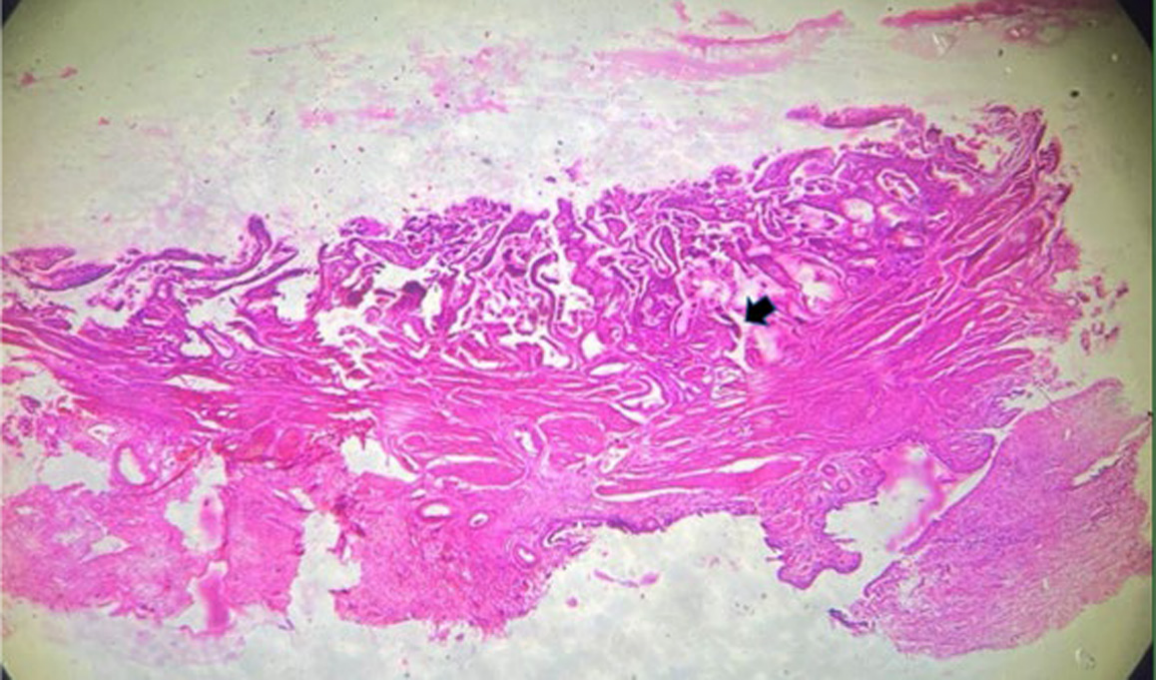
Histopathology of specimen shows chronic cholecystitis. Patient underwent full recovery, and on follow‐up the patient is doing well.

The patient's postoperative recovery was smooth and uneventful. Histopathological examination of the cholecystectomy specimen revealed significant findings. The tissue showed papillary columnar mucosal lining forming Rokitansky–Aschoff sinuses, which are associated with chronic cholecystitis. There was dense lymphonuclear infiltrate extending to the underlying hyalinizing muscularis propria and congested serosal layer, confirming a diagnosis of chronic regenerative cholecystitis (Figure 6). Postoperatively, the patient was closely monitored and showed no recurrence of symptoms or complications. The case was managed effectively through accurate diagnostic imaging and appropriate surgical intervention, demonstrating the complexity of biliary anomalies and the importance of precise diagnosis in guiding treatment.

## 5 DISCUSSION

This case presents a novel and rare instance of a duplicate gallbladder anomaly, which is a significant deviation from more commonly encountered biliary conditions. The diagnosis of a duplicate gallbladder, characterized by the presence of two separate gallbladders with individual cystic ducts, is extraordinarily rare and provides a unique opportunity to discuss the complexities and diagnostic challenges associated with such anomalies. The patient's presentation with severe right upper quadrant pain, nausea, vomiting, and weight loss initially pointed towards common biliary disorders like choledochal cysts or cholecystitis. However, advanced imaging ultimately revealed the rare condition of a duplicate gallbladder, underscoring the critical role of detailed imaging in diagnosing complex biliary conditions.

The rarity of duplicate gallbladders makes this case particularly noteworthy. The majority of documented cases are discovered incidentally during imaging or surgical procedures performed for other conditions.^3^ For instance, Tiwari and Sharma reported a case of duplicate gallbladder found during laparoscopic cholecystectomy, but the imaging characteristics and detailed preoperative evaluation were not as comprehensive as those presented in this case.^3^ Similarly, Singh et al. described a case involving a duplicate gallbladder where MRCP played a crucial role in diagnosis; however, their report lacked the detailed imaging sequence and differential diagnostic approach demonstrated here.^6^ This case stands out due to the use of both MRCP and 3D ultrasound to provide a thorough evaluation of the biliary anatomy, which allowed for the precise identification of the duplicate gallbladder and its anatomical relationship with the common bile duct.

Choledochal cysts are a more common differential diagnosis and are characterized by congenital dilations of the bile duct. They often present with symptoms such as abdominal pain, jaundice, and, in severe cases, cholangitis or pancreatitis.^1^ The imaging features of choledochal cysts typically include cystic dilations of the bile duct and are classified into different types based on their anatomical location and extent. Choledochal cysts are often diagnosed in childhood but can occasionally present in adults with atypical symptoms, which can complicate the diagnosis and make distinguishing between similar conditions challenging.^2^

In this case, MRCP provided critical information by revealing a thin‐walled cystic lesion adjacent to the gallbladder that communicated with the bile duct and contained multiple calculi. The MRCP images were instrumental in differentiating this lesion from a choledochal cyst. The detailed imaging allowed visualization of the cyst's communication with the gallbladder and clarified the anatomical relationship between the gallbladder and the common bile duct, which was essential for accurate diagnosis and surgical planning.^4^ The additional use of 3D ultrasound confirmed these findings and demonstrated a thin communicating tract between the gallbladder and the common bile duct, effectively ruling out choledochal cysts and confirming the presence of a duplicate gallbladder.^5^

The surgical findings corroborated the imaging results, revealing a double gallbladder Y‐type anomaly. The extrahepatic gallbladder was distended and filled with clear mucus, while the intrahepatic gallbladder contained multiple stones. The cystic duct, which was short and straight, joined directly with the common bile duct. The identification of two cystic arteries and the presence of thick fibrofatty tissue at the hilum suggested acute chronic inflammation, providing further evidence of the complex nature of this condition.^7^

The detailed imaging and surgical exploration in this case highlight the importance of high‐resolution imaging techniques in diagnosing rare biliary anomalies. Accurate MRCP and 3D ultrasound evaluations allowed for the differentiation of a duplicate gallbladder from more common conditions such as choledochal cysts. This precise diagnostic approach is essential for planning effective surgical interventions and avoiding unnecessary procedures or misdiagnoses.

This case contributes valuable insights into the understanding and management of duplicate gallbladder anomalies. It demonstrates how advanced imaging can improve diagnostic accuracy and guide effective treatment strategies in managing complex biliary conditions. The integration of MRCP and 3D ultrasound in diagnosing this rare anomaly underscores the necessity of detailed imaging in the assessment of biliary disorders, enhancing our ability to manage and treat such uncommon conditions effectively.

## 6 CONCLUSION

This case of a duplicate gallbladder underscores the critical role of advanced imaging techniques in diagnosing rare and complex biliary anomalies. The use of MRCP and 3D ultrasound was instrumental in accurately identifying the duplicate gallbladder and differentiating it from other biliary disorders, such as choledochal cysts. This precise diagnostic approach not only facilitated appropriate surgical planning but also highlighted the necessity of thorough imaging in managing unusual biliary conditions. The case demonstrates that detailed and accurate imaging can significantly enhance diagnostic accuracy and improve patient outcomes by guiding effective treatment strategies for rare biliary anomalies.

## AUTHOR CONTRIBUTIONS

**Momin Shakeeb:** Conceptualization; writing – original draft. **Romana Riyaz:** Conceptualization; writing – original draft; writing – review and editing. **Sajjad Ahmed Khan:** Conceptualization; writing – original draft; writing – review and editing. **Khadija Fatima:** Writing – original draft. **Husna Fatima:** Writing – original draft.

## FUNDING INFORMATION

None.

## CONFLICT OF INTEREST STATEMENT

The authors have no conflict of interest to declare.

## CONSENT

Written informed consent was obtained from the patient to publish this report in accordance with the journal's patient consent policy.

## Data Availability

Data will be provided by the corresponding author upon reasonable request. Images uploaded in the separate files.
